# Erfahrungen mit der digitalen Versorgung von Patienten mit chronischen und akuten Lungenerkrankungen während der SARS-CoV-2-Pandemie

**DOI:** 10.1007/s00108-022-01266-3

**Published:** 2022-02-18

**Authors:** Marcel Braun, Olaf Schmidt, Thomas Schultz, Holger Woehrle, Martina Große Sundrup, Christoph Schöbel

**Affiliations:** 1 Universitätsmedizin Essen, Ruhrlandklinik – Westdeutsches Lungenzentrum, Klinik für Pneumologie, Lehrstuhl für Schlaf- und Telemedizin, Universität Duisburg-Essen, Essen, Deutschland; 2 KPPK Studienzentrum Koblenz/Pneumologische Gemeinschaftspraxis Koblenz, Koblenz, Deutschland; 3 PneumologenLichterfelde Berlin, Berlin, Deutschland; 4 Pneumologische VersorgungsForschung e. V. (PVF), Berlin, Deutschland; 5 Lungenzentrum Ulm, Ulm, Deutschland; 6 Ruhrlandklinik, Westdeutsches Lungenzentrum am Universitätsklinikum Essen gGmbH, Universitätsmedizin Essen, Tüschener Weg 40, 45239 Essen, Deutschland

**Keywords:** Asthma bronchiale, Chronisch-obstruktive Lungenerkrankung, Von Patienten berichtete Erfahrungen, Telemonitoring, Spirometrie, Asthma, Pulmonary disease, chronic obstructive, Patient-reported experience, Remote monitoring, Spirometry

## Abstract

**Hintergrund:**

Das Management von Patienten mit respiratorischen Erkrankungen ist seit Beginn der Severe-acute-respiratory-syndrome-coronavirus-2(SARS-CoV-2)-Pandemie durch Maßnahmen zur Infektionsprävention teilweise erschwert. Um die Versorgung zu gewährleisten, wurde ein digitales Versorgungsprogramm implementiert, mit dem Krankheitsverläufe von Patienten per Smartphone-App und Bluetooth-Spirometer überwacht werden können.

**Zielsetzung:**

Ermittlung der Erfahrungen von Patienten und Ärzten mit der digitalen Versorgung.

**Material und Methoden:**

Zur Analyse der Erfahrungen wurden strukturierte Fragebögen entwickelt, mit denen verschiedene Dimensionen aus Sicht von Patienten und Ärzten bewertet wurden. Nutzung und Interaktion wurden quantitativ erfasst.

**Ergebnisse:**

In das Programm wurden 745 Patienten mit Asthma, chronisch-obstruktiver Lungenerkrankung, Zustand nach „coronavirus disease 2019“ (COVID-19) sowie anderen Lungenerkrankungen eingeschlossen. Im mittleren Nachsorgezeitraum von 49,4 ± 12,6 Wochen erfolgten durchschnittlich 289 Messungen. Aus Patientenperspektive wurde das digitale Programm positiv bewertet: Die höchste Bewertung wurde für „Erfahrung mit der häuslichen Messung“ ermittelt (1,4 ± 0,5; 99 % positiv), gefolgt von „Kommunikation/Interaktion“ (1,8 ± 0,9; 83 % positiv). Ein Anteil von 70 % gab eine subjektive Verbesserung der Lebensqualität durch die Teilnahme am Programm an. Die Bewertung durch die Ärzte war mit einer mittleren Bewertung von 2,2 ± 1,2 ebenfalls positiv.

**Schlussfolgerung:**

Das App-basierte digitale Versorgungsprogramm konnte sinnvoll in die Routineversorgung während der SARS-CoV-2-Pandemie integriert werden und hat das Potenzial, die Versorgung auch darüber hinaus zu unterstützen. Patientenrelevante Erfahrungen sind in allen Dimensionen positiv und die digitale Versorgung wurde gut akzeptiert. Vonseiten der teilnehmenden Ärzte wird das Programm positiv bewertet, was sich an der hohen Interaktion mit der Plattform und den positiven Bewertungen der Effekte zeigt.

**Zusatzmaterial online:**

Die Online-Version dieses Beitrags (10.1007/s00108-022-01266-3) enthält eine Übersicht der teilnehmenden Studienzentren.

## Abstract (en)

**Background:**

Management of patients with respiratory disorders, such as asthma or chronic obstructive pulmonary disease (COPD), became challenging during the severe acute respiratory syndrome coronavirus 2 (SARS-CoV-2) pandemic due to infection prevention measures. To maintain care, a remote monitoring program was initiated, comprising a smartphone app and a Bluetooth spirometry device.

**Objective:**

To assess patient- and physician-related experience with remote monitoring.

**Material and methods:**

Structured questionnaires were developed to rate experiences from the patient or physician perspective on six-level Likert scales. Interactions between patients and physicians via the digital platform and overall utilization was analyzed.

**Results:**

A total of 745 patients with asthma, COPD, post-coronavirus disease 2019 (COVID-19) and other respiratory diseases were enrolled from 31 centers in Germany. Mean follow-up was 49.4 ± 12.6 weeks. Each participant submitted on average 289 measurements. Patient-reported experience with the remote monitoring program was positive, with the highest satisfaction reported for “Experience with home measurement” (1.4 ± 0.5; 99% positive), followed by “Communication/interaction” (1.8 ± 0.9; 83% positive) and “Overall satisfaction with program” (1.8 ± 0.8; 87% positive). In all, 70% reported subjective quality of life improvements related to participation in the program. Physician satisfaction with the program was also high with a mean rating of 2.2 ± 1.2.

**Discussion:**

App-based remote monitoring was successfully implemented in routine care during the SARS-CoV‑2 pandemic and demonstrated potential for improvements in care. Patient-relevant experience was positive in all dimensions and remote monitoring was well accepted. Physicians who participated in the program also expressed positive experiences, as demonstrated by a high level of interaction with the platform and positive evaluations of effects from the program.

Asthma bronchiale und die chronisch-obstruktive Lungenerkrankung („chronic obstructive pulmonary disease“ [COPD]) gehören zu den häufigsten pneumologischen Erkrankungen und betreffen über 400 Mio. Menschen weltweit [1, 2]. Trotz fortschreitender Verbesserung präventiver Maßnahmen und therapeutischer Interventionen bedingen diese Erkrankungen weiterhin eine hohe Morbidität, welche die Lebensqualität der Betroffenen erheblich einschränken und insbesondere bei unzureichender Kontrolle häufige Hospitalisierungen notwendig machen kann. Durch die seit März 2020 fortschreitende Severe-acute-respiratory-syndrome-coronavirus-2(SARS-CoV-2)-Pandemie sind diese Patienten zusätzlich dem hohen Risiko einer schweren „coronavirus disease 2019“ (COVID-19) ausgesetzt. Wiederholt eingesetzte Maßnahmen zur Infektionsprophylaxe führen zudem zu einer erheblichen Reduktion der Kontakte zwischen Patienten und Ärzten, wodurch sich die Behandlung dieser Erkrankungen verschlechtern kann. So zeigen Daten aus verschiedenen geografischen Regionen eine deutliche Reduktion der Inanspruchnahme medizinischer Leistungen, sowohl im allgemein- als auch im fachärztlichen Bereich [3–5].

Bereits vor der SARS-CoV-2-Pandemie wurden zunehmend digitale Versorgungskonzepte eingesetzt. Diese haben jedoch seit Frühjahr 2020 erheblich an Bedeutung gewonnen und sind inzwischen integraler Bestandteil vieler Gesundheitssysteme. Insbesondere App-basierte Programme, die Patienten auf ihren Mobiltelefonen installieren und die diese mit ihren Behandlern direkt verbinden, haben das Potenzial, die Gesundheitsversorgung nachhaltig und positiv zu beeinflussen. So können indikations- bzw. therapiespezifische Applikationen das Management von Behandlungen unterstützen, mögliche Versorgungslücken schließen sowie die Wirksamkeit von Interventionen verbessern. Vernetzte Messverfahren, mit denen Patienten selbstständig physiologische Parameter erheben, und die Übertragung der Daten über Mobiltelefone an den behandelnden Arzt ermöglichen zudem das kontinuierliche Monitoring des Gesundheitszustands ohne physischen Kontakt in der Klinik oder Praxis. Dies kann insbesondere bei chronischen Erkrankungen, wie Diabetes, arterieller Hypertonie oder Asthma, positive Versorgungseffekte erzielen und die Kosteneffektivität von Behandlungen verbessern [6]. So zeigten sich in Studien und Metaanalysen Verbesserungen in der Adhärenz, im Selbstmanagement und der Lebensqualität, aber auch prozessuale Effekte im Sinne schnellerer Diagnostik und höherer Termintreue [7–11].

Patientenberichtete Erfahrungen sind ein Indikator für die Qualität medizinischer Maßnahmen

Da bei diesen Technologien der Nutzen häufig nicht über klassische Endpunkte ermittelt werden kann, gewinnt die Erhebung von Daten zu subjektiven patienten- bzw. providerrelevanten Erfahrungen an Bedeutung. So fordern Leitlinien zur Bewertung der Evidenz telemedizinischer Interventionen, dass neben patientenberichteten Wirksamkeitsendpunkten („patient-reported outcome measures“ [PROM]), wie der Veränderung der gesundheitsbezogenen Lebensqualität, auch die patientenberichteten Erfahrungen („patient-reported experience measures“ [PREM]) in die Ermittlung des Nutzens einbezogen werden sollen [12, 13]. PREM erfassen dabei gestützt auf strukturierte Fragebögen Erfahrungen, die ein Patient mit einem Diagnostik- bzw. Behandlungsprozess gemacht hat, und stellen so einen Indikator für die Qualität der Maßnahmen dar [14].

Zur Gewährleistung der Versorgung von Patienten mit chronischen respiratorischen Erkrankungen während der SARS-CoV-2-Pandemie wurde im März 2020 ein bundesweites Projekt zwischen Leistungserbringern aus der Pneumologie und Industriepartnern initiiert. Dieses basiert auf einem App-gestützten digitalen Versorgungskonzept für Patienten mit pneumologischen Erkrankungen, das bereits 2018 in einer randomisierten, kontrollierten Studie an einem kleineren Kollektiv klinisch geprüft wurde [15]. Zur Evaluation des Projekts im Hinblick auf den Nutzen in der Routineversorgung wurde eine wissenschaftliche Auswertung implementiert, deren Ergebnisse in diesem Beitrag vorgestellt werden sollen.

## Material und Methoden

### Digitale Versorgung

**Figure 1:**
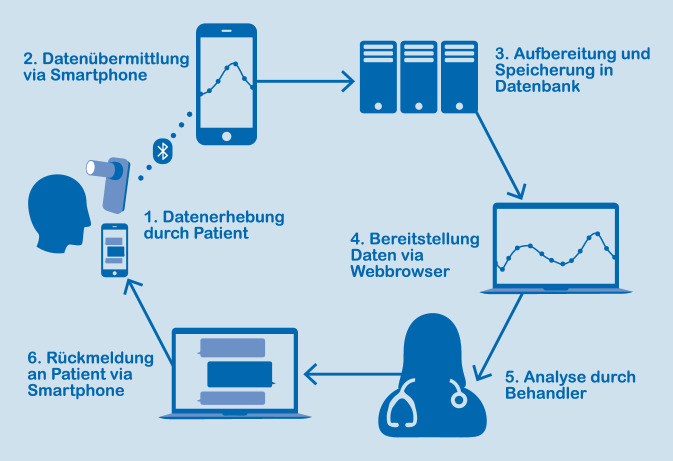

**Figure 2:**
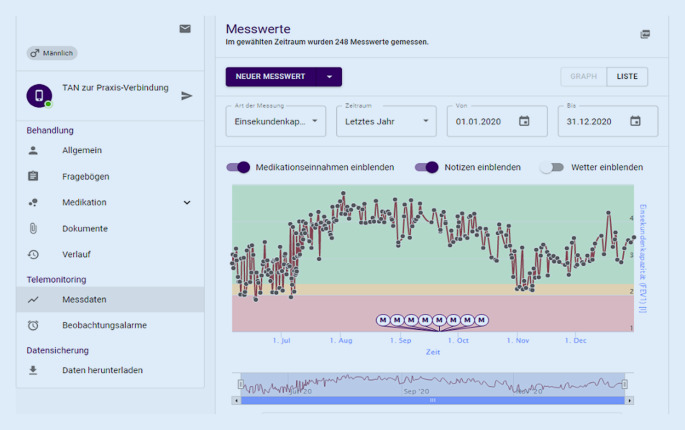

Mithilfe einer Gesundheits-App (SaniQ, Qurasoft GmbH, Koblenz), die speziell für Patienten mit Lungenerkrankungen entwickelt wurde, konnten Teilnehmer verschiedene Parameter digital aufzeichnen und an behandelnde Ärzte übermitteln sowie bei Bedarf direkt mit ihrem behandelnden Arzt über Nachrichten kommunizieren (Abb. 1). Teilnehmende Patienten installierten die App auf ihren Mobiltelefonen und erhielten ein Spirometer, das mittels Bluetooth die Werte an die Telefone überträgt (Smart One, Medical International Research Inc, Rom, Italien). Patienten konnten weitere analog erhobene Messungen, wie das Gewicht, oder auch die eingenommene Medikation in der App speichern. Studienzentren (Tab. S1 im elektronischen Zusatzmaterial online) erhielten Zugriff auf die Daten der von ihnen eingeschriebenen Patienten über eine webbasierte Plattform (SaniQ Praxis, Qurasoft GmbH, Koblenz), mit der so ein umfangreiches Telemonitoring ermöglicht wurde (Abb. 2). Über die Plattform für Studienzentren konnten zudem Fragebögen, beispielsweise zur gesundheitsbezogenen Lebensqualität, direkt an die eingeschlossenen Patienten gesendet und ausgewertet werden.

### Wissenschaftliche Evaluation

Zur Ermittlung des Nutzens der App-gestützten digitalen Versorgung in der klinischen Routine wurde, nach Einholen eines positiven Ethikvotums (Ethikkommission, Universitätsmedizin Essen), eine einarmige Observationsstudie durchgeführt. In die Studie eingeschlossen wurden Patienten mit

- Asthma bronchiale (Global-Initiative-for-Asthma[GINA]-Stadium III–IV, mit und ohne antiasthmatische Antikörpertherapie),
- COPD (Global-Initiative-for-Chronic-Obstructive-Lung-Disease[GOLD]-Stadium II–IV, mit und ohne Sauerstofftherapie),
- Zustand nach COVID-19-Erkrankung sowie
- anderen Lungenerkrankungen, bei denen ein Telemonitoring sinnvoll erschien.

Ziel der Studie war, Nutzen und Erfahrungen mit der App-gestützten digitalen Versorgung zu evaluieren, insbesondere vor dem Hintergrund, die Versorgung mittels Telemonitoring und digitaler Kommunikation während der SARS-CoV-2-Pandemie außerhalb ambulanter und stationärer Einrichtungen durchführen zu können.

Zur Analyse des Nutzungsverhaltens und der Interaktion über die Gesundheits-App wurden Daten über die webbasierte Plattform des Herstellers quantitativ erfasst. Die Bestimmung des Nutzens der Gesundheits-App und patientenrelevanter Erfahrungen erfolgte mit einem strukturierten Fragebogen, der auf Basis eines bereits validierten Instruments entwickelt wurde [16]. Dieses Instrument wurde gewählt, da die abgefragten Dimensionen eine hohe Übereinstimmung mit den für dieses Projekt zu beantwortenden Fragestellungen aufwiesen. Eine gewisse Adaptation war jedoch notwendig, um der heterogenen Patientenpopulation und der verwendeten Monitoringtechnologie gerecht zu werden. Der Fragebogen wurde direkt über die Gesundheits-App an alle Teilnehmer versendet und ausgefüllt. Er umfasste 17 Items in den drei Dimensionen „Erfahrung mit der häuslichen Messung“, „Kommunikation/Interaktion“ sowie „Bewertung des Programms“, zu denen jeweils das Ausmaß der individuellen Bewertung auf einer 6‑stufigen Likert-Skala von 1 = „stimme voll und ganz zu“ bis 6 = „stimme überhaupt nicht zu“ angegeben wurde.

Zur Ermittlung des Nutzens aus Sicht der Leistungserbringer wurde ein programmspezifischer strukturierter Fragebogen entwickelt, der 13 Items in 8 Dimensionen umfasste („usability“, Koordination der Behandlungsabläufe, Ausrichtung an Leitlinien und Standards, Adhärenz, Patientensicherheit, Gesundheitskompetenz, Smartphone-basierte Anwendung allgemein, technische Unterstützung). Die Bewertung erfolgte ebenfalls mittels 6‑stufiger Likert-Skala, analog zum Fragebogen für Patienten. Der Fragebogen wurde von der Studienleitung über eine digitale Plattform an die teilnehmenden Studienzentren übermittelt und online ausgefüllt.

Die Auswertung erfolgte mittels deskriptiver und schließender Statistik. Zur Validierung wurden die interne Konsistenz mittels Cronbachs α sowie die Inter-Item-Korrelationen bestimmt.

## Ergebnisse

### Patientenpopulation

An der Studie nahmen über einen Zeitraum von 12 Monaten bundesweit 31 Studienzentren (25 ambulante und 6 stationäre Einrichtungen) teil, aus denen insgesamt 745 Patienten mit Asthma, COPD, Zustand nach SARS-CoV-2-Infektion mit COVID-19-Erkrankung sowie anderen Lungenerkrankungen rekrutiert wurden (Tab. 1). Der Nachsorgezeitraum betrug im Mittel 49,4 ± 12,6 Wochen. Teilnehmende Patienten waren durchschnittlich 47,4 ± 15,5 Jahre alt (14–80 Jahre) und in der Mehrzahl weiblich (58,8 %).

**Table 1:** 

	Gesamt (*N* = 745)	Männlich (*n* = 315)	Weiblich (*n* = 430)	*p*-Wert (m vs. w)
Alter (Jahre, Mittelwert ± SD)	47,4 ± 15,5	49,2 ± 15,6	46,1 ± 15,2	0,007
Geschlecht (m/w, %)	42,3/57,7	n.a.	n.a.	n.a.
Body-Mass-Index (Mittelwert ± SD, kg/m^2^)	27,0 ± 6,0	27,4 ± 5,1	26,7 ± 6,5	0,003
*Diagnose (%)*				
Asthma	43,6	44,9	42,8	n. s.
COPD	2,0	2,2	1,9	n. s.
COVID-19	6,6	7,3	6,0	n. s.
Andere/unbekannt	47,8	45,5	49,1	n. s.

*COPD* „chronic obstructive pulmonary disease“ (chronisch-obstruktive Lungenerkrankung), *COVID-19* „coronavirus disease 2019“, *m* männlich, *n.a.* nicht anwendbar, *n.s.* nicht signifikant, *SD* „standard deviation“ (Standardabweichung), *w* weiblich

### Messadhärenz und Kommunikation

Während des Studienzeitraums wurden im Mittel 289 Messungen pro Teilnehmer mit dem Bluetooth-Spirometer durchgeführt und über die Gesundheits-App an die Studienzentren übermittelt („peak expiratory flow“ [PEF]: 106.768 Messungen, 143 ± 208 pro Teilnehmer; forciertes exspiratorisches Volumen [FEV_1_]: 109.030 Messungen, 146 ± 208 pro Teilnehmer; Tab. 2). Die Messadhärenz, definiert als mindestens eine Messung pro Monat bzw. pro Woche, betrug 65,8 und 48,5 %.

**Table 2:** 

	Insgesamt	Pro Patient	Weiblich	Männlich	*p*-Wert (m vs. w)
Messungen PEF	106.768	143 ± 208	146 ± 223	139 ± 185	n. s.
Messungen FEV_1_	109.030	146 ± 208	149 ± 225	142 ± 182	n. s.
*Kommunikation*					
An Arzt gesendete Nachrichten	1501	2,0 ± 4,3	1,9 ± 4,0	2,1 ± 4,7	n. s.
An Patienten gesendete Nachrichten	1777	2 ± 3	2 ± 3	2 ± 3	n. s.

*FEV*_*1*_ forciertes exspiratorisches Volumen, *m* männlich, *n.s.* nicht signifikant, *PEF* „peak expiratory flow“, *w* weiblich

Teilnehmende Patienten versendeten insgesamt 1501 Nachrichten an die Studienzentren (2,0 ± 4,3 pro Teilnehmer), während von Ärzten in den Studienzentren 1777 Nachrichten an teilnehmende Patienten übermittelt wurden (2,0 ± 4,3 pro Teilnehmer). Medizinische Variablen wie Alter, Geschlecht oder Grunderkrankung hatten keinen Einfluss auf das Ausmaß der Kommunikation über die Gesundheits-App. Bei Patienten mit Asthma zeigte sich eine moderate negative Korrelation zwischen der Anzahl der gesendeten Nachrichten und dem durchschnittlichen Asthma-Quality-of-Life-Questionnaire(AQLQ)-Wert über den Studienzeitraum (*r* = −0,267, *p* =0,009, *n* = 95).

### Patientenberichtete Erfahrungen

Von 745 Patienten, die in die Studie eingeschlossen wurden, nahmen 231 an der Befragung zu patientenrelevanten Erfahrungen mit der Gesundheits-App teil (31 %). Patienten, die an der Befragung teilnahmen, waren signifikant älter (*p* < 0,001), unterschieden sich darüber hinaus jedoch nicht und waren somit repräsentativ für die Studienpopulation. Die interne Konsistenz der Skalen des Fragebogens wurde mittels Cronbachs α bestimmt und mit einem Wert von 0,847 und akzeptablen Inter-Item-Korrelationen als ausreichend erachtet.

**Figure 3:**
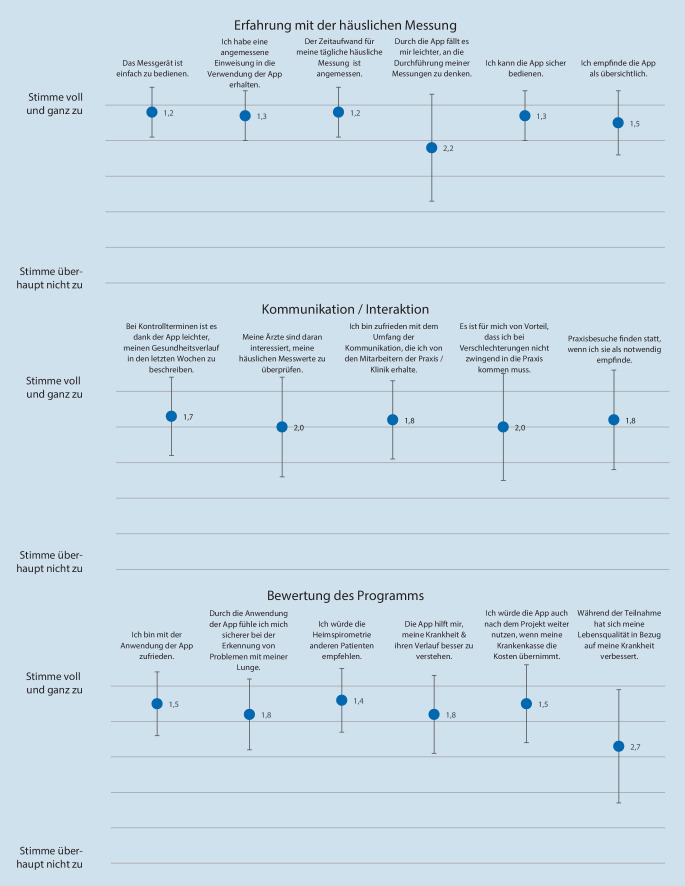

Die Gesamtbewertungen waren positiv und ergaben mittlere Werte von 1,4 ± 0,5 (99 % positive Einschätzung, positiv definiert als Wert zwischen 1 und 3 auf Likert-Skala) für „Erfahrung mit der häuslichen Messung“, 1,8 ± 0,9 (83 % positive Einschätzung) für „Kommunikation/Interaktion“ sowie 1,8 ± 0,8 (87 % positive Einschätzung) für „Bewertung des Programms“. Innerhalb der Dimensionen wichen einige Items deutlich von der jeweiligen Gesamtbewertung ab (Abb. 3). In der Dimension „Erfahrung mit der häuslichen Messung“ war dies das Item „Durch die Smartphone-basierte Anwendung fällt es mir leichter, an die Durchführung meiner Messungen zu denken“ (mittlere Zustimmung 2,2 ± 1,5), und in der Dimension „Bewertung des Programms“ das Item „Während der Teilnahme hat sich meine Lebensqualität in Bezug auf meine Krankheit verbessert“ (mittlere Zustimmung 2,7 ± 1,6). Die Einschätzung dieses Items korrelierte dabei signifikant mit der Gesamtbewertung des Versorgungsprogramms (*r*_*s*_ = 0,679, *p* < 0,001, *n* = 227). Die weitere Analyse zeigte eine positive Einschätzung dieses Items bei 70 % der Studienteilnehmer. Von den teilnehmenden Patienten gaben 83 % an, die Smartphone-basierte Anwendung nach Studienende weiter nutzen zu wollen, wenn die Krankenversicherung die Kosten übernähme. Lediglich 4,3 % lehnten eine weitere Nutzung ab.

Medizinische Variablen wie Alter, Body-Mass-Index oder Grunderkrankung hatten keinen signifikanten Einfluss auf die Bewertung der einzelnen Dimensionen oder auf die Gesamteinschätzung des Nutzens des Programms.

### Erfahrungen von Ärzten

Die Nutzung der webbasierten Plattform durch die behandelnden Ärzte blieb über den Studienzeitraum konstant hoch, insgesamt wurden 4626 Zugriffe registriert (51,4 ± 96,5 pro Zentrum). Pro Woche erfolgten pro Studienzentrum im Mittel 2,8 Zugriffe auf die mittels Telemonitoring übermittelten Daten.

**Figure 4:**
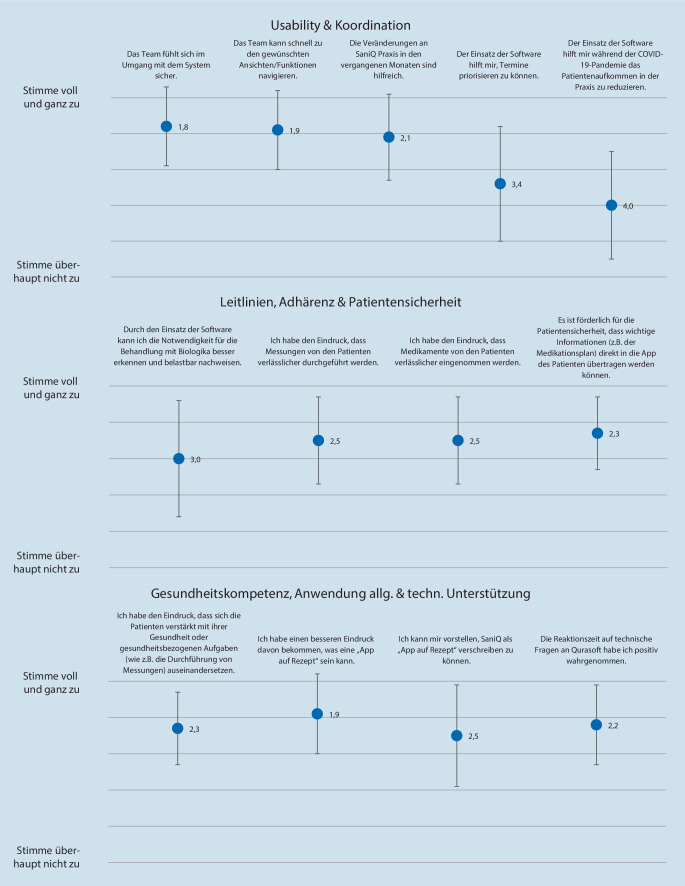

Von 31 Studienzentren nahmen 27 an der abschließenden Befragung zu providerrelevanten Erfahrungen teil (87 %). Der Nutzen der App-gestützten digitalen Versorgung wurde von den teilnehmenden Studienärzten insgesamt mit einer mittleren Bewertung von 2,2 ± 1,2 positiv eingeschätzt (Abb. 4). Positiv wichen die Dimensionen „usability“ sowie „technische Unterstützung“ ab (1,9 ± 0,8 bzw. 1,6 ± 0,7). Negative Abweichungen zeigten sich in den Dimensionen „Koordination der Behandlungsabläufe“ sowie „Ausrichtung an Leitlinien und Standards“ (3,7 ± 1,5 bzw. 3,0 ± 1,6).

## Diskussion

Aktuell werden in Deutschland zunehmend digitale Gesundheitsanwendungen (DiGA) zugelassen und in die Versorgung eingeführt [17]. Stand 02/2022 wurden bislang 30 DiGA als erstattungsfähig zugelassen, die größtenteils Indikationen aus dem neurologisch-psychiatrischen Spektrum abdecken oder z. B. bei onkologischen Erkrankungen unterstützend eingesetzt werden [18]. Bislang wurde keine DiGA für Patienten mit pneumologischen Erkrankungen zugelassen, obwohl hier insbesondere bei Asthma, COPD oder auch in der respiratorischen Schlafmedizin aufgrund der chronischen Verläufe ein erhebliches Potenzial besteht, die Versorgung zu verbessern [19].

Die Patienten bewerteten das Versorgungsprogramm durchweg positiv

Mit den hier vorgestellten Ergebnissen liegen nun erste Daten zu Erfahrungen von Patienten und Providern mit einer Gesundheits-App für das Telemonitoring bei respiratorischen Erkrankungen in der Routineversorgung vor. Insgesamt zeigt sich eine gute Akzeptanz der App-gestützten digitalen Versorgung aufseiten beteiligter Patienten und behandelnder Ärzte. Patienten bewerteten das Versorgungsprogramm durchweg positiv, wobei insbesondere die Interaktion mit der digitalen Technologie und die Teilnahme am Programm insgesamt hohe Zustimmung erhielten. Positive Versorgungseffekte bestehen in einer verbesserten Gesundheitskompetenz und einer hohen Adhärenz bei Lungenfunktionsmessungen. Zudem berichteten immerhin 70 % der Patienten Lebensqualitätsverbesserungen durch die Teilnahme am digitalen Versorgungsprogramm, sodass auch hieraus ein relevanter Zusatznutzen entsteht. Mögliches Potenzial der Technologie besteht weiter in der Früherkennung von Exazerbationen durch die Dynamik der übermittelten Lungenfunktionswerte sowie in der Zunahme der Kommunikation zwischen Patient und Arzt. So zeigte sich eine signifikant häufigere Kommunikation bei Patienten mit schlechteren durchschnittlichen AQLQ-Werten im Studienzeitraum (*p* = 0,009), was für eine stärkere Interaktion bei erhöhtem medizinischem Bedarf spricht. Ein weiterer Nutzen der Gesundheits-App zeigt sich in der hohen Adhärenz bei Spirometermessungen von 65,8 % mit mindestens einer übermittelten Messung pro Monat. Gegenüber der Routineversorgung, bei der maximal 1‑mal pro Quartal eine Messung in der Praxis durchgeführt wird, lassen sich Verschlechterungen so deutlich früher erkennen und Korrekturmaßnahmen einleiten.

Die hier vorgestellten Ergebnisse zur App-gestützten digitalen Versorgung von Patienten mit respiratorischen Erkrankungen in der klinischen Routine bestätigen frühere Untersuchungen an kleineren Kollektiven [15]. Diese konnten ebenfalls deutliche Effekte aus Patientenperspektive im Hinblick auf Verbesserungen von Lebensqualität und Krankheitskontrolle in einem randomisierten, kontrollierten Studiendesign nachweisen.

Vonseiten teilnehmender Ärzte wird die App-gestützte digitale Versorgung ebenfalls positiv bewertet, was sich an einer hohen Interaktion mit der Plattform und an positiven Bewertungen der Effekte durch das Programm zeigt. Als positive Effekte werden insbesondere die verbesserte Mess- und Medikamentenadhärenz sowie insgesamt eine Zunahme der Gesundheitskompetenz in Bezug auf die Erkrankung genannt. Verbesserungspotenzial besteht aus Sicht der Studienzentren in der Integration der digitalen Technologie in den Praxis- bzw. Klinikalltag. Wichtig erscheint, dass innerhalb der Praxen bzw. Kliniken entsprechende Prozesse etabliert werden, mit denen eingehende Daten sachgerecht und effizient verarbeitet werden können. Mit Zunahme der Datenqualität und -menge lassen sich zukünftig möglicherweise durch Verwendung von Algorithmen oder künstlicher Intelligenz Daten automatisiert bzw. teilautomatisiert verarbeiten. Auch die Einbindung von geschultem medizinischem Assistenzpersonal oder von telemedizinischen Zentren kann möglicherweise helfen, Prozesse effizienter zu gestalten. Des Weiteren muss eine entsprechende Vergütungsstruktur für diese neuen Versorgungsformen etabliert werden.

Neben der großen Anzahl an Studienzentren und Teilnehmern sind die Ergebnisse der Studie relevant, da hiermit erstmals ein eindeutig positiver Effekt durch eine Gesundheits-App auf die Versorgung von Patienten mit Lungenerkrankungen in Deutschland nachgewiesen werden konnte. Daten aus dem Ausland zeigen eine ähnliche Tendenz, wobei die Ergebnisse teilweise inkonsistent sind und sich nicht immer ein klarer Effekt ableiten lässt [20–22].

### Limitationen

Aufgrund der Durchführung in der klinischen Routine weist die Studie einige relevante Limitationen auf. Zunächst ließ das Studiendesign keinen Vorher-nachher-Vergleich eingeschlossener Patienten zu. Da das Versorgungsprogramm zu Beginn der SARS-CoV-2-Pandemie innerhalb kurzer Zeit implementiert wurde, war es nicht möglich, systematische Eingangsuntersuchungen bei eingeschlossenen Patienten durchzuführen. Entsprechend dem primären Ziel, Patienten während der Pandemie mittels Telemonitoring zu unterstützen, wurde zudem auf die Einrichtung einer Kontrollgruppe verzichtet. Auch wenn prinzipiell ein kontrolliertes Design möglich wäre, ließ die Situation im März 2020 dies nicht zu, da die Zielpopulation aufgrund der Lungenerkrankungen bereits ein erhöhtes Risiko einer COVID-19-Erkrankung aufwies und Patienten keiner weiteren Unsicherheit durch Randomisierung ausgesetzt werden sollten. Um das Potenzial einer Technologie vollumfänglich abschätzen zu können, ist neben der Prüfung in kontrollierten Studien jedoch auch die Evaluation von Effekten in der klinischen Routine wichtig, sodass die Studie hier relevante Erkenntnisgewinne liefert.

## Schlussfolgerung

Insgesamt zeigen sich positive Effekte und Erfahrungen mit der untersuchten App-gestützten digitalen Versorgung aufseiten betroffener Patienten und behandelnder Ärzte. Es besteht zwar noch Optimierungsbedarf hinsichtlich der Anpassung von Behandlungsabläufen auf Grundlage standardisierter Interventionsbeschreibungen und hinsichtlich der Integration der digitalen Kommunikation in die klinische Versorgung. Potenzial für eine Verbesserung der Versorgung durch den Einsatz der Gesundheits-App bei Patienten mit chronischen Lungenerkrankungen ist aber erkennbar, was sich auch aus der hohen Bereitschaft zur weiteren Teilnahme an dem Programm ergibt.

## Fazit für die Praxis

Digitale Technologien gewinnen im Gesundheitswesen weiter an Bedeutung. Das hier vorgestellte Versorgungsprogramm zeigt, dass ein App-basiertes Monitoring bei Patienten mit Lungenerkrankungen sinnvoll und zügig in die Routine von Praxen und Kliniken integriert werden kann. Patientenrelevante Erfahrungen sind positiv und führen mehrheitlich zu Verbesserungen der Lebensqualität durch Teilnahme am digitalen Versorgungsprogramm. Die beteiligten Ärzte schätzen die Technologie ebenfalls positiv ein, wobei Optimierungspotenzial bei der Integration in existierende Prozesse sowie bei der Finanzierung der digitalen Versorgung besteht.

## Supplementary Information




