# Supplementary material for: Experiences with digital care of patients with chronic and acute lung diseases during the SARS-CoV-2 pandemic
Source: Internist (Berl). 2022 Feb 18;63(3):255–65. [Article in German] doi: 10.1007/s00108-022-01266-3 (PMC8856116; doi:10.1007/s00108-022-01266-3)
Supplement: Supplementary file 1 [file 108_2022_1266_MOESM1_ESM.docx]

Supplement zu

**Erfahrungen mit der digitalen Versorgung von Patienten mit chronischen und akuten Lungenerkrankungen während der SARS-Cov-2 Pandemie**

Braun M ^1^, Schmidt O ^2^, Schultz T ^3, 4^, Woehrle H ^5^, Große Sundrup M ^1^, Schöbel C ^1^

^1^ Universität Duisburg-Essen, Universitätsmedizin Essen, Ruhrlandklinik - Westdeutsches Lungenzentrum, Klinik für Pneumologie, Lehrstuhl für Schlaf- und Telemedizin

^2^ KPPK Studienzentrum Koblenz / Pneumologische Gemeinschaftspraxis Koblenz

^3^ PneumologenLichterfelde Berlin

^4^ PVF Pneumologische Versorgungsforschung e.V. Berlin

^5^ Lungenzentrum Ulm

**Teilnehmende Studienzentren**

| **Studienzentrum** | **Stadt** | **Studienleiter** | **N** |
| --- | --- | --- | --- |
| Ärztehaus St. Hedwig Kaiserslautern | Kaiserslautern | Prof. Dr. Michael Schmitz | 87 |
| Dipl.-Med. Anne-Kathrin Schmidt | Oschatz | Anne-Kathrin Schmidt | 21 |
| Dipl.-Med. Marion Duenzel | Niesky | Marion Dünzel | 1 |
| Gemeinschaftspraxis Dr. Schmidt, Frau Dipl.-Med. Gronke | Dresden | Dr. Frank Schmidt | 5 |
| Gemeinschaftspraxis Raubach | Raubach | Dr. Ute Bettig | 20 |
| Gemeinschaftspraxis Reinfeld | Reinfeld | Dr. Frank Kannies | 44 |
| Internistische Gemeinschaftspraxis Bitburg - Trier | Trier | Dr. Patrick Albrecht | 11 |
| Lungenärzte Radebeul | Radebeul | Dr. Rudolf Hennig | 1 |
| Lungenpraxis | Konstanz | Dr. Maren Schuhmann | 3 |
| Lungenpraxis Dr. Susanne Ziebuhr und Dr. Frank Richter | Plauen | Dr. Susanne Ziebuhr | 4 |
| Lungenzentrum Essen | Essen | Dr. Tilmann Genz | 12 |
| Lungenzentrum Ulm | Ulm | Holger Woehrle | 20 |
| Lungenzentrum Worms | Worms | Dr. Max Apprich | 21 |
| Medizinische Hochschule Hannover | Hannover | PD Dr. Hendrik Suhling | 13 |
| Pneumologen Lichterfelde | Berlin | Dr. Thomas Schultz | 62 |
| Pneumologie Markendorf | Dresden | Dr.Andre Markendorf | 2 |
| Pneumologische Gemeinschaftspraxis Koblenz | Koblenz | Dr. Olaf Schmidt | 135 |
| Pneumologische Praxis Gauting | Gauting | Dr. Stefan Heindl | 11 |
| Pneumologische Praxis PD Dr. Geßner | Leipzig | PD Dr. Christian Geßner | 3 |
| Praxis Dr. Antje Hammers-Reinhard | Homburg-  Saar | Dr. Antje  Hammers-Reinhard | 8 |
| Praxis Dr. Hans-Christian Blum | Dortmund | Dr. Hans-Christian Blum | 13 |
| Praxis Dr. Holger Hein | Reinbeck | Dr. Holger Hein | 20 |
| Praxis Dr. med. Arne Drews & Dtefan Bartmuß | Grimma | Dr. Arne Drews | 42 |
| Praxisgemeinschaft Dr. Abenhardt / Hinrichs-Pavlik | Heidelberg | Dr. Birgit Abenhardt | 23 |
| Praxisgemeinschaft Dr. Abenhardt / Hinrichs-Pavlik | Heidelberg | Dr. Jochen Hinrichs-Pavlik | 10 |
| Rhein-Maas-Klinikum | Würselen | Dr. Peter Fleimisch | 1 |
| Ruhrlandklinik Essen | Essen | Prof. Dr. Christoph Schöbel | 71 |
| Schön Klinik Berchtesgadener Land | Schönau am Königssee | Prof. Rembert Koczulla | 39 |
| Schwerpunktpraxis Lungen- und Bronchialheilkunde, Allergologie und Schlafmedizin | Düsseldorf | Dr. Charles Lange | 20 |
| Universitätsklinikum Hamburg-Eppendorf | Hamburg | PD Dr. Hans Klose | 2 |
| Universitätsklinikum Magdeburg | Magdeburg | Dr. Monique Vorsprach | 21 |
